# Detachable and Reusable: Reinforced π‐Ion Film for Modular Synaptic Reservoir Computing

**DOI:** 10.1002/adma.202506729

**Published:** 2025-06-27

**Authors:** Gyu Won Woo, Chang Min Lee, Won Woo Lee, Min Ju Jung, Seung Min Lee, Hye Won Lee, Hocheon Yoo, Yong Hee Kim, Eun Kwang Lee

**Affiliations:** ^1^ Department of Chemical Engineering Pukyong National University Busan 48513 Republic of Korea; ^2^ Department of Artificail Intelligence Semiconductor Engineering Hanyang University Seoul 04763 Republic of Korea; ^3^ Department of Electronic Engineering Hanyang University Seoul 04763 Republic of Korea

**Keywords:** detachable electronics, neuromorphic reservoir computing, organic electrochemical transistors, synaptic devices, π‐ion film

## Abstract

Organic electrochemical transistors (OECTs) show significant promise for bioelectronics and neuromorphic computing applications due to their low operating voltage, biocompatibility, and ion‐mediated charge transport. However, conventional OECTs with permanently fixed organic semiconductor (OSC) layers lack modularity and reusability for sustainable electronics with e‐waste reduction. Here, a novel reinforced π‐ion film OECT featuring a detachable and reusable OSC layer that creates a unified composite with dielectric and gate components, establishing a new paradigm for modular device architectures is proposed. Through solvent exchange and mesh‐supported gelation, π‐ion film exhibits enhanced mechanical stability, detachability, and superior electrical performance. The OECTs demonstrate remarkable 35‐day air stability, 50‐day storage lifetime, and over 80% performance retention after 600 electrical cycles. Furthermore, the π‐ion film OECTs exhibit synaptic behavior with paired‐pulse facilitation of 167% and long‐term memory retention of 34% maintained synaptic current after 250 s. These characteristics enable reservoir computing applications with a 4‐bit encoding scheme for image recognition, processing 16 × 16 pixelated input patterns, demonstrating reliable state differentiation and stable signal retention. Even at lab‐scale development, reinforced π‐ion film OECTs represent a promising eco‐friendly platform for modular, reusable components in next‐generation neuromorphic computing systems, aligning with electronic waste reduction policies by enabling component reuse.

## Graphical abstract

This study presents a reinforced π‐ion film for organic electrochemical transistors (OECTs), addressing the rapid degradation of organic semiconductor layers. By introducing a mesh support and utilizing a scalable solvent exchange method, the π‐ion film enhances detachability and stability. The resulting OECTs demonstrate superior performance and synaptic properties, paving the way for sustainable, modular electronic systems.

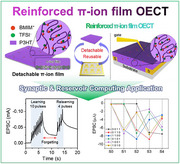

## 1 Introduction

Recently, advances in organic semiconductors (OSCs) have enabled the development of flexible and biocompatible devices with potential applications in wearables,^[^ ^1^, ^2^, ^3^, ^4^, ^5^, ^6^, ^7^ ^]^ displays,^[^ ^8^, ^9^, ^10^, ^11^, ^12^ ^]^ and electronics with neuromorphic computing.^[^ ^13^, ^14^, ^15^, ^16^, ^17^ ^]^ The evolution of OSC‐based electronic devices has progressed through three generations: 1) organic field‐effect transistors (OFETs), 2) organic electrochemical transistors (OECTs), and 3) reinforced π‐ion film OECTs. While OFETs rely on gate field‐effect amplification at high operating voltages,^[^ ^18^ ^]^ OECTs offer significant advantages for neuromorphic and bioelectronic applications due to their low operating voltage (≈1 V) and ability to mimic biological synapses.^[^ ^19^, ^20^, ^21^, ^22^, ^23^ ^]^ OECTs function via ion transport in the ion gel, where the applied gate voltage (*V*_GS_) facilitates ion movement, leading to charge doping in the OSC channel.^[^ ^24^, ^25^, ^26^ ^]^ Previously, J. Ji *et* al. introduced a pulse‐driven synaptic electrochemical transistor that achieved twice higher pH sensitivity of 124 mV pH^−1^ than the Nernstian limit of 59.2 mV pH^−1^ and 7169 times faster response of 8.75 ms than the potentiostatic sensors of 62.73 s by optimizing the gate bias near the transconductance intersection, effectively mimicking neuromorphic functions for enhanced biosensor performance.^[^ ^27^ ^]^ This neuromorphic approach also showed minimizations to calibration fluctuations and power consumption, paving the way for standardized, high‐performance biosensors. Furthermore, M. Wu *et* al. developed a stretchable, skin‐integrated neuromorphic system using triboelectric nanogenerators for tactile sensing and OECTs for information processing.^[^ ^28^ ^]^ This system demonstrated key neuromorphic functions, including sensitive pressure detection (≈0.04 kPa^−1^), long‐term synaptic plasticity, and high mechanical durability of 100% strain, enabling effective artificial tactile perception for wearable applications.

However, OECTs face a critical challenge; the OSC layer undergoes rapid performance degradation due to repeated ion injections and exposure to ambient conditions, particularly charge trapping by oxygen and water. This instability leads to reduced operational performance^[^ ^29^ ^]^ and air stability^[^ ^30^ ^]^ compared to more robust electronic components such as metal electrodes and insulators. Addressing this limitation requires a multifaceted approach integrating material design,^[^ ^31^ ^]^ stabilizer addition,^[^ ^32^ ^]^ and encapsulation.^[^ ^33^ ^]^ On the materials front, the development of conjugated polymers with intrinsically stable backbones and hydrophobic side chains has demonstrated promise in mitigating environmental degradation.^[^ ^34^ ^]^ Beyond material innovation, interface engineering is delicate yet effective in stabilizing OECT performance. Controlling the electrolyte‐semiconductor interaction through tailored surface treatments^[^ ^35^ ^]^ or self‐assembled monolayers^[^ ^36^ ^]^ can suppress parasitic charge trapping and enhance operational durability. Additionally, the use of electrolyte formulations with optimized ion mobility and minimal side reactions can further improve device longevity.^[^ ^37^, ^38^ ^]^ At the device level, strategies such as dynamic biasing and pulsed operation have been explored to mitigate the cumulative effects of charge trapping and ion‐induced degradation.^[^ ^39^ ^]^ By optimizing the electrical stress profile during operation, these approaches can extend the device's lifetime while preserving performance metrics such as transconductance and switching speed.

While conventional approaches address OSC degradation through frequent device replacement, a more sustainable solution involves a modular OECT design that allows selective replacement of the OSC layer without compromising device performance. The modular OECT design provides a sustainable alternative by extending the device lifespan, reducing material waste, and lowering maintenance costs. Moreover, stable performance across multiple detachment cycles ensures long‐term reliability, making this approach both practical and scalable for bioelectronic and neuromorphic applications. Nevertheless, achieving a reliable, detachable, and replaceable OSC layer presents several challenges: i) Conventional OSC films suffer from poor mechanical integrity, making them prone to structural damage during handling. ii) Additionally, their low stiffness and weak interfacial adhesion often lead to delamination, cracking, or performance degradation upon detachment and reattachment.

To address the limitations of rapid OSC degradation, in this work, a detachable and modular OECT platform using reinforced π‐ion films was introduced. This approach enables selective replacement of the OSC layer while ensuring stable electrical performance over multiple detachment and reattachment cycles. The π‐ion film, formed by methanol (MeOH) solvent exchange and subsequent ionic liquid (IL) injection, was reinforced with mesh support to enhance mechanical integrity. A key innovation in this approach is the conceptualization of the OECT as a unified 1‐composite system where the OSC: dielectric (electrolyte): gate components are considered holistically rather than as discrete elements. This integrated perspective allows for strategic reinforcement at critical interfaces while maintaining essential electro‐ionic interactions. The reinforced π‐ion film serves as the cornerstone of this composite vision, preserving functionality across multiple assembly cycles. Compared to conventional OSC films, this reinforced π‐ion film exhibited improved structural robustness, allowing reliable handling without delamination or performance degradation. By integrating this reinforced π‐ion film into OECTs, enhanced operating stability, air stability, and shelf life while preserving neuromorphic functionality were achieved. The device demonstrated reusability across multiple detachment/attachment cycles without significant electrical deterioration, offering a sustainable alternative to conventional OECTs. Additionally, synaptic properties of the OECTs were validated by implementing key neuromorphic applications, including Pavlovian learning and image recognition using 4‐bit reservoir computing. This modular, reconfigurable design not only extends the device lifespan but also facilitates adaptive and energy‐efficient bioelectronic applications, marking a step forward in the development of next‐generation synaptic electronics. The π‐ion film‐based OECTs herein can give all the potential applications of existing OECT devices for neuromorphic functions, reservoir computing and logical gates. In addition, the π‐ion film‐based OECTs have novel new concepts with detachable and reusable properties, replaceability after large‐area manufacturing, and modularity.

The modular OECT platform presented herein aligns with increasingly stringent electronic waste (e‐waste) management policies being implemented globally. By enabling selective replacement of only the degraded OSC layer rather than discarding entire devices, this approach significantly reduces electronic component waste and extends the functional lifetime of semiconductor‐based technologies. This design philosophy represents a paradigm shift in sustainable electronics, where componentization and modular design principles are applied at the microscale. This paradigm extends beyond academic research, offering a blueprint for the industrial scale‐up of sustainable electronic systems with extended operational lifespans.

## 2 Results and Discussion

### 2.1 Material Characterization of the π‐Ion Film

**Figure 1:**
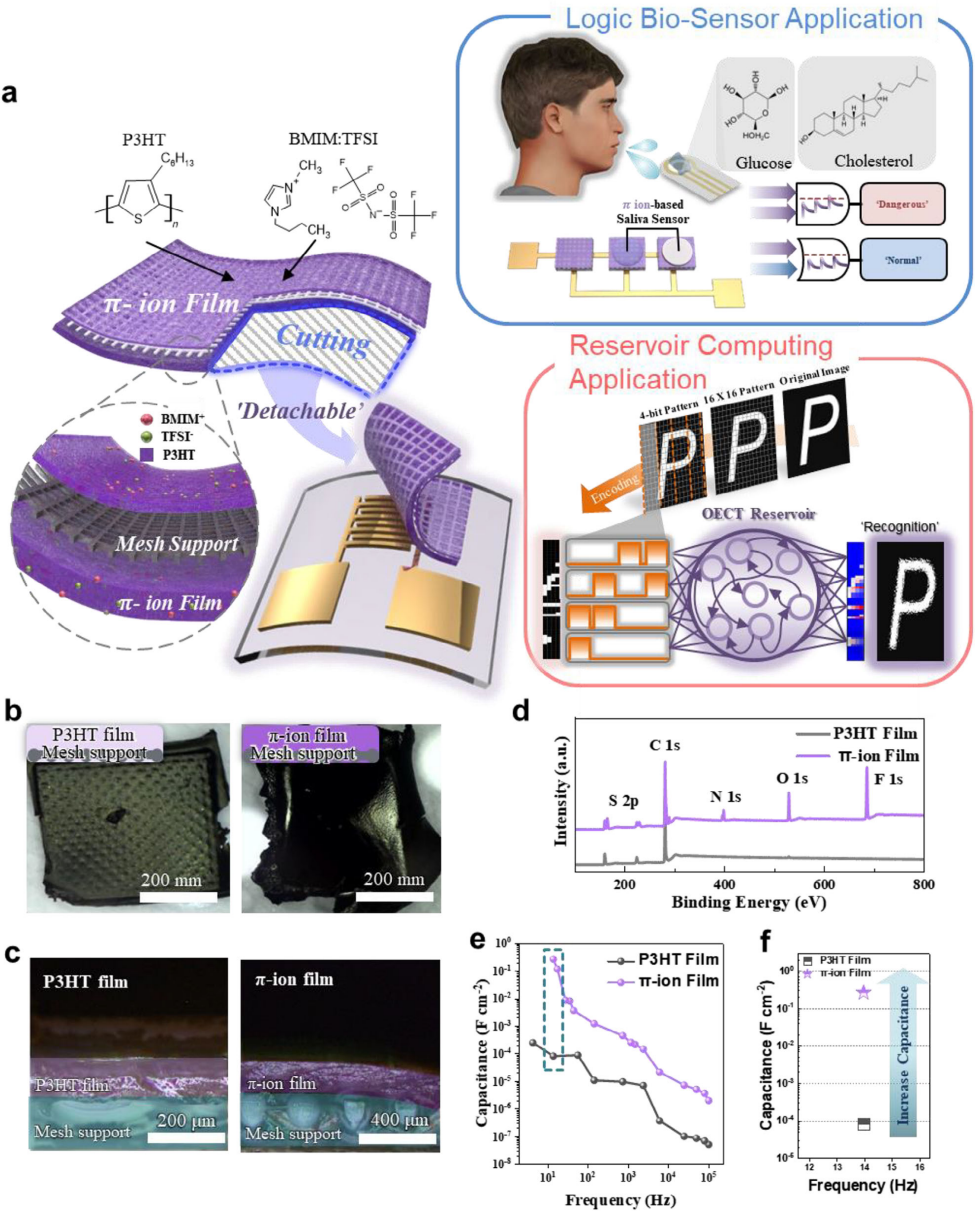
Reinforced π‐ion film. a) Detachable and reusable reinforced π‐ion film for modular synaptic reservoir computing and possibility of biosensor application. b) Photo images and c) cross‐sectional OM images of pristine P3HT film (left) and π‐ion film (right). d) XPS spectra showing S and C peaks of P3HT in both films, while N, O, and F peaks from the IL only in the pi‐ion film, demonstrating uniform π‐ion film fabrication. e,f) Capacitance from EIS measurements indicating the π‐ion film exhibited a significantly higher capacitance than pristine P3HT film.

The π‐ion films used in this study were fabricated using a solvent exchange‐driven gelation method, inspired by previous studies on poly(para‐phenyleneethynylene)s (PPEs)‐based π‐ion gels.^[^ ^40^, ^41^ ^]^ In this method, a solution of the OSC in a good solvent (e.g., Tetrahydrofuran, THF) undergoes controlled diffusion of a poor solvent (e.g., MeOH), inducing aggregation of polymer chains via π–π stacking interactions. In our system, P3HT and [BMIM][TFSI] were used as the OSC and the IL. These differ from the PPEs and [TBMA][TFSI] combination reported by Kushida et al.; the same mechanism applies.^[^ ^41^ ^]^ A volume ratio of 4:1 (P3HT solution: IL) was used, based on the optimal range identified in previous studies. This ratio ensures that the polymer network remains continuous while providing sufficient ionic pathways through the gelled ionic liquid matrix. **Figure** **1**a shows a fabricated device with π‐Ion film gel made by combining P3HT and 1‐Butyl‐3‐methylimidazolium bis(trifluoromethylsulfonyl)imide (BMIM:TFSI), which can be cut, attached and detached from an electrode substrate using the mechanically reinforcing effect of mesh support. Also, the mesh support is made of conductive stainless steel and serves as the gate electrode in π‐Ion film‐based OECTs. The π‐Ion film is a single component that acts as an OSC, dielectric, and gate in OECT. In this study, π‐Ion film‐based OECTs showed reservoir computing applications based on synapse characteristics and potential applications of biosensor logic for health diagnosis. Figure 1b shows photo images of the pristine P3HT film and the π‐ion film on mesh support, showing the thicker thickness of the π‐ion film. The cross‐sectional optical microscope (OM) images (Figure 1c) revealed that the pristine P3HT film exhibited a thickness of ≈120 µm, whereas the π‐ion film was fabricated at ≈200 µm thickness. The increased thickness is attributed to the introduction of IL, which facilitates the formation of the gel structure and contributes to the substantial material layer required for electronic device applications. Comparing the surface morphology of pristine P3HT film and the π‐ion film through top‐view OM images (Figure S2a, Supporting Information) demonstrated that the BMIM:TFSI IL was uniformly embedded into the P3HT polymer matrix, which is essential for achieving a consistent film structure for uniform electrical performance. The scanning electron microscope (SEM) images (Figure S2b, Supporting Information) further confirmed the constant IL distribution within the P3HT chains, suggesting that the integration of the IL enhanced the π‐ion film's structural integrity and uniformity as the previous study.^[^ ^41^ ^]^ The interaction between the π‐ion film and the substrate is primarily governed by van der Waals forces. The π‐ion film contains an embedded ionic liquid within its structure, forming a gel‐like network with the π‐conjugated polymer. Compared to a purely solid‐state film, this ion liquid‐based structure offers enhanced adhesion to the substrate, owing to its partial fluidity and better surface conformity as Figure S3 (Supporting Information) showing adhesion properties after even 7 days. Additionally, the long‐term structural stability of the π‐ion film can be attributed to the use of solvent‐promoted gelation, rather than thermally‐promoted gelation, during polymer preparation. This approach results in a more stable gel network. The film's structural integrity is further reinforced by a solvent exchange process, which stabilizes the gel as described in previous study.^[^ ^40^ ^]^

Figure 1d shows the chemical composition of the pristine P3HT and π‐ion films using X‐ray photoelectron spectroscopy (XPS). The presence of peaks corresponding to sulfur (S) 2p and carbon (C) 1s in both films confirmed the dominance of P3HT. Additionally, the appearance of nitrogen (N) 1s, oxygen (O) 1s, and fluorine (F) 1s peaks in the π‐ion film indicated the successful incorporation of the BMIM:TFSI IL in the P3HT matrix.

Electrochemical impedance spectroscopy (EIS) measurements, shown in Figure 1e, demonstrated the charge transport mechanism within the π‐ion film. The capacitance from the EIS measurements at the low‐frequency region from 10 to 100 Hz, is known as the ion‐induced charge transport mechanism region for OECT applications.^[^ ^36^ ^]^ When comparing the capacitance between the π‐ion film and pristine BMIM:TFSI IL in the low‐frequency region (Figure 1f), the capacitances were 117.52 and 0.08 mF cm^−2^, respectively. The π‐ion film exhibited a significantly higher capacitance, suggesting that ion polarization induced by the ion trap interaction between P3HT chains and BMIM:TFSI ions within the π‐ion film were more efficient, which can induce the high‐performance electrical performance of OECTs and enhanced the ion trapping effects for synaptic behavior in the pulse *V*_GS_.

### 2.2 Electrical Performance of π‐Ion Film/Mesh OECTs

**Figure 2:**
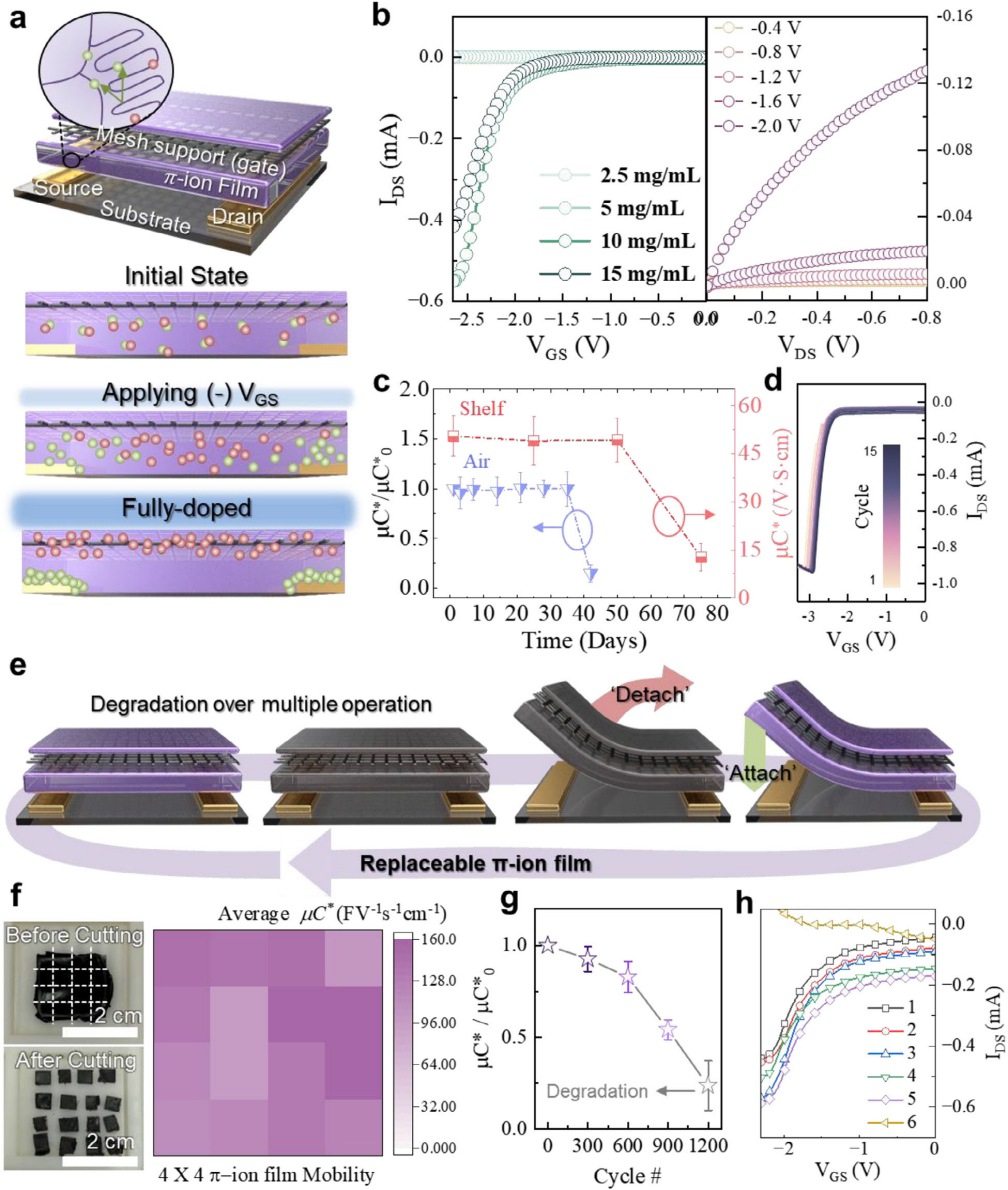
Electrical and detachable characteristic of π‐ion film‐based OECT. a) Device structure and doping mechanism of the π‐ion film‐based OECT. b) Transfer curves (left) of π‐ion film/mesh‐based OECT according to P3HT concentrations, showing the highest electrical performance at 10 mg mL^−1^ and declined performance at 15 mg mL^−1^ due to excessive concentration (*V*_DS_ = −1 V). Output curves (right) of π‐ion film/mesh‐based OECT with P3HT concentration of 10 mg mL^−1^. c) Air stability (blue) and shelf life (red) of π‐ion film/mesh‐based OECT showing no electrical performance degradation for 35 days in air and 50 days in storage condition (temperature of 25 °C and humidity of 25%). d) 15 cycles transfer curves of π‐ion film/mesh‐based OECT showing operation stability (*V*_DS_ = −1 V). e) Schematic illustration of replacement of the detachable π‐ion film functionalized by mesh support for enabling continued device use. f) Images of the large‐area film cut into a 4 × 4 array (left) and electrical performance of the corresponding cut films (right). g) Electrical performance of π‐ion film/mesh OECT according to operation cycle after replacement of a π‐ion film. h) Transfer curves showing consistent electrical performance after multiple detaching/attaching cycles of the same π‐ion film.

The device structure of the π‐ion film/mesh‐based OECT was illustrated at the top of **Figure** **2**a. In *p*‐type operation, the extent of TFSI anions intercalation into P3HT modulates conductivity in response to negative *V*_GS_. As shown in the bottom of Figure 2a, the operation mechanism of the device through doping was explained. When a negative voltage is applied to the gate (stainless steel mesh support), the TFSI anions in π‐ion film migrate toward the source‐drain electrodes, forming an electric double layer and channel area near the source‐drain electrodes, while the BMIM cations move toward the gate. This process forms ohmic contact between the π‐ion film and the electrodes, inducing *p*‐type doping in P3HT to increase conductivity.^[^ ^41^ ^]^

To determine the optimal P3HT concentration, transfer curves were measured at various P3HT concentrations from 2.5 to 15 mg mL^−1^ (Figure 2b, left). The highest electrical performance showing the OECT mobility (µC^*^, where *μ* is mobility and *C* ^*^ is volumetric capacitance) of 97.25 F V^−1^ s^−1^ cm^−1^ was observed at a concentration of 10 mg mL^−1^. However, when the concentration increased beyond 10 mg mL^−1^, electrical performance degradation occurred due to two main factors: First, the high concentration of the OSC relative to the IL resulting in deficient OSC doping, which hindered effective charge transport. Second, the increased film thickness leads to a reduced *V*_GS_ effect and decreased µC^*^. Conversely, when the concentration of P3HT was lower than 10 mg mL⁻¹, the density of the polymer chains was insufficient to form a continuous and interconnected crystalline network within the film. This insufficient molecular ordering prevented the formation of π–π stacked domains that provide effective charge transport pathways, resulting in poor electrical performance despite gate voltage application.^[^ ^42^, ^43^ ^]^ The stable output characteristics of the optimized π‐ion film/mesh OECTs were shown on the right side of Figure 2b. In Figure 2d, transfer curves over 15 cycles confirmed the operational stability of the devices. The π‐ion film/mesh structure‐based OECTs exhibited a low *V*_th_ of −2.31 V, along with high transconductance (*g*_m_) and µC^*^ of 3.88 mS and 206.80 F V^−1^ s^−1^ cm^−1^, respectively. This demonstrates superior electrical performance compared to that of the P3HT π‐film and electrolyte structure OECTs, which showed 0.23 mS and 6.05 F V^−1^ s^−1^ cm^−1^ (**Table** **1**; Figure S4, Supporting Information). This improvement can be attributed to the integration of the electrolyte and OSC, which reduced the ion migration distance and increased µC^*^. Additionally, the presence of mesh support made of conductive stainless steel allowed for the decreased *V*_th_, enhanced µC^*^ and *g*_m_. Figure S4a (Supporting Information) shows the P3HT π‐film device without ionic liquid, representing a solid‐state π‐ion film. The thickness of the P3HT π‐film is approximately 1 µm, resulting in a longer ion transport pathway compared to conventional P3HT thin‐film OECTs. Consequently, the device requires a higher operating *V*_GS_ up to −6 V to effectively inject ions into the active channel. Figure S5 (Supporting Information) shows the gate leakage current (*I*_GS_) induced by the electrolyte BMIM:TFSI within the channel which not only facilitates effective ion transport but also contributes to a controlled leakage current.

**Table 1:** Electrical performance comparison of P3HT π‐film/electrolyte, π‐ion film, and π‐ion film/mesh‐based OECTs.

Structure	*g*_m, max_ [mS]^a)^	*V*_th_ [V]^b)^	*I*_on_/*I*_off_ ^c)^	µC* [F V^−1^ s^−1^ cm^−1^]^d)^
P3HT π‐film/electrolyte	0.23 (±0.03)^e)^	−2.87 (±0.20)	43.60 (±6.08)	6.05 (±0.91)
π‐ion film	1.84 (±0.50)	−2.46 (±0.04)	5.64 (±1.79)	179.50 (±25.22)
π‐ion film/mesh	3.88 (±0.41)	−2.31 (±0.05)	30.00 (±5.58)	206.80 (±40.94)

^a)^ *g*_m,max_: Maximum transconductance;

^b)^ *V*_th_: Threshold voltage;

^c)^ *I*_on_/*I*_off_: On/Off ratio;

^d)^ µC*: Charge carrier mobility of OECTs;

^e)^ The 5 devices were measured for statistical analysis (*V*_DS_ = –1 V and *W*/*L* = 10).

Figure 2c shows the air stability and shelf life of the π‐ion film/mesh OECTs, demonstrating no significant degradation in electrical performance up to 35 days, with the µC^*^ change compared to the initial day (µC^*^_0_) due to the passivation effect of IL encapsulated on P3HT. This high stability of π‐ion film/mesh OECTs could be utilized as components of sensors that could be damaged in polluted environments of air, operating for 30 days before π‐ion film/mesh replacement. Furthermore, electrical performances after storing the device in storage conditions (temperature: 24.5 °C, humidity: 25%) for 25, 50, and 75 days were measured. The π‐ion film/mesh OECTs maintained stable performance even after 50 days of storage. The actual storage environment is shown in Figure S6 (Supporting Information). These results show that the device exhibits stable performance over a long period. This stability can be attributed to the addition of IL, which helps maintain a moist state in the gelation phase and serves as a passivation role to minimize the impact of external environmental factors, thereby enhancing performance stability. For comparison, the air stability (temperature: 25 °C, humidity: 25%) of spin‐coated P3HT‐based conventional OECTs was analyzed (Figure S7, Supporting Information). The µC^*^ of pristine P3HT decreased to 20% in 2 days due to charge trapping by water and oxygen in the air. On the other hand, the above P3HT π‐ion film in Figure 2c showed high air stability for up to 35 days due to the passivation effect of the ion gel on P3HT molecules. **Table** **2** demonstrates the superior electrical performance, reusability and detachability of our device with a simplified structure compared to previously reported P3HT‐based OECTs.

**Table 2:** Summary table of P3HT‐based OECTs for key electrical parameter, stability, functionality characteristic.

Year	Device Structure	Channel	Electrolyte	µC* [F V^−1^s^−1^cm^−1^]	*g*_m, max_ (mS)	Stability [cycle]	Reusability	Detachability	Application	Refs.
2025	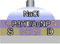	AuNP/ P3HT	NaCl(aq)	97.55 (±10.2)	>0.7	40	X	X	N/A	[51]
2025	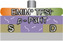	p‐P3HT (cross‐linked)	[EMIM]+ [TFSI]‐(gel)	≈30	12	100	X	X	N/A	[52]
2024	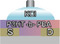	P3HT‐b‐PBA	KCl (aq)	170 (±40.9)	≈0.17	110	X	X	N/A	[53]
2024	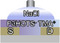	P3HOTS‐TMA+	NaCl(aq)	1.38	0.86	630	X	X	N/A	[54]
2019	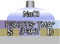	PTHS‐ TMA+ ‐co‐ P3HT	NaCl(aq)	1.7	≈0.42	N/A	X	X	N/A	[55]
2025	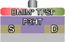	P3HT	[BMIM]+ [TFSI]‐ (gel)	161 (± 31)	4.8 (±3.9)	N/A	X	X	N/A	[56]
2025	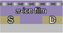	π‐ion film (P3HT‐based)	[BMIM]+ [TFSI]‐	206.8 (±40.94)	3.88 (±0.4)	600	O	O	Reservoir computing, Bio‐sensor	This work

### 2.3 Detachable and Modulable Characteristics

The introduction of mesh support significantly enhanced the detachability and reusability of the π‐ion film. This mesh support structure allows the film to be fixed on the support, providing the functionality for easy detachment and reattachment. This functionality mechanism is shown in Figure 2e. Because of the physical stability provided by the mesh support, large‐area films can be fabricated and cut to desired sizes for practical applications including modularity for replacement of OSC channel layers which typically exhibit relatively rapid performance degradation. By utilizing the detachable characteristics of the π‐ion film, it is possible to replace only the OSC channel layer, allowing for continuous use of the device. The left side of Figure 2f shows the large‐area film (2 cm × 2 cm size) cut into a 4 × 4 array (0.5 cm × 0.5 cm size), the process of cutting the fabricated film for device production. This practical aspect emphasizes the potential to create modular components tailored to specific requirements. The capacity to cut the film is enabled by the mesh support, which effectively stabilizes the π‐ion film, ensuring that the film maintains its structure even when the mechanical stress was applied with the cutting process. The right side of Figure 2f shows the comparison of µC^*^ metrics for each segment. The comparison showed that each segment exhibited a uniform average µC^*^ of 131.32 F V^−1^ s^−1^ cm^−1^, allowing for uniform functionality after replacement, which is a crucial factor in overcoming the rapid performance decline typically associated with OSCs. A video of the cutting and electrical performance measurement process was shown in Figure S8 (Supporting Information). The operation stability of the OECTs after attachment is presented in Figure 2g. The devices maintained over 80% electrical performance stability after 600 operation cycles, confirming the reliability of the π‐ion film's electrical stability. This finding demonstrated the potential for long‐term use of the devices by resolving the inherent degradation of OSC materials. Figure 2h indicates the stability of electrical performance under the multiple replacement process of the detachable π‐ion film. This represented the operation stability up to 5 cycles, with *I*_DS_ increase due to the accumulation of doping effects from repeated measurements and different gate contact areas. A degradation in electrical performance after the sixth cycle is attributed to mechanical mismatch and interfacial strain accumulated during repeated detachment, as shown in Figure S9 (Supporting Information). This may lead to partial delamination and disruption of polymer crystallinity, affecting charge transport. However, the π‐ion film maintains reliable performance over multiple cycles, confirming its suitability for moderate reuse ≈5 times. Images of the mismatched film after repeated replacement is provided in Figure S9a–g (Supporting Information). In addition, the other reason the detachable π‐ion film device fails to turn on after the sixth replacement cycle is the loss of ions from the π‐ion film matrix, as Figure S9h (Supporting Information). Repeated manual detachment causes scratches and mechanical stress on the film surface. Over multiple cycles, these accumulated scratches allow the ionic liquid which is originally embedded within the gel matrix to leak out. The resulting loss of ions, which are essential for active layer operation, ultimately leads to device failure. For comparison, the same multiple replacement processes were conducted on the π‐ion gel without the mesh support. In this case, electrical performance degradation was observed immediately after the second measurement following detachment (Figure S10a, Supporting Information). Thus, the mesh support plays a crucial role in maintaining the structural integrity of the π‐ion film, thereby facilitating the effective detaching and attaching process of the π‐ion film. Additionally, to assess the physical stability and durability of the film during the detachment process, OM images were taken before and after placing the film on the source‐drain electrodes demonstrating that the film could be detached cleanly, with existing particles adhering to the π‐ion film and being removed along with it (Figure S10b,c, Supporting Information). To demonstrate the scalability of the π‐ion film platform, a large‐area array consisting of 64 devices was fabricated on a 4 × 4 cm^2^ substrate by utilizing its cuttable and modular properties (Figure S11, Supporting Information). Consistent electrical performance of transfer characteristics from 16 randomly selected transistors in the array was measured, confirming its potential for integration into scalable and reconfigurable array systems (Figure S12, Supporting Information). These results indicate that the fabricated π‐ion film can be applied to various device forms and customized depending on the shape and application of the devices. Figure S13 (Supporting Information) shows the higher operation stability and lower hysteresis phenomenon of π‐ion film/mesh‐based OECTs compared to spin‐coated P3HT at drain voltage *(V*_DS_) of −1 V over 15 cycles. In the π‐ion film, ions are already embedded and mobile within the P3HT channel matrix. On the other hand, in the conventional OECT structure, ions reside in a separate phase and must penetrate through an interface to reach the channel. During repeated cycling, it is crucial not only that ions can efficiently enter the active channel but also that they can be smoothly extracted. In the conventional OECT structure where ions originate from a separate phase, this interface can impede ion extraction, particularly after multiple cycles, leading to performance degradation. By contrast, the π‐ion film, which contains ions embedded directly within the channel matrix, allows for more reversible and unhindered ion transport. These advantages are key factors contributing to the superior cycling stability and low hysteresis observed in π‐ion film‐based devices. The integration of mesh support in the π‐ion film/mesh OECTs not only enables modularity and reusability but also enhances the overall stability and versatility of the device, addressing issues related to OSC materials.

### 2.4 Synaptic Behavior Analysis and Application in Reservoir Computing

**Figure 3:**
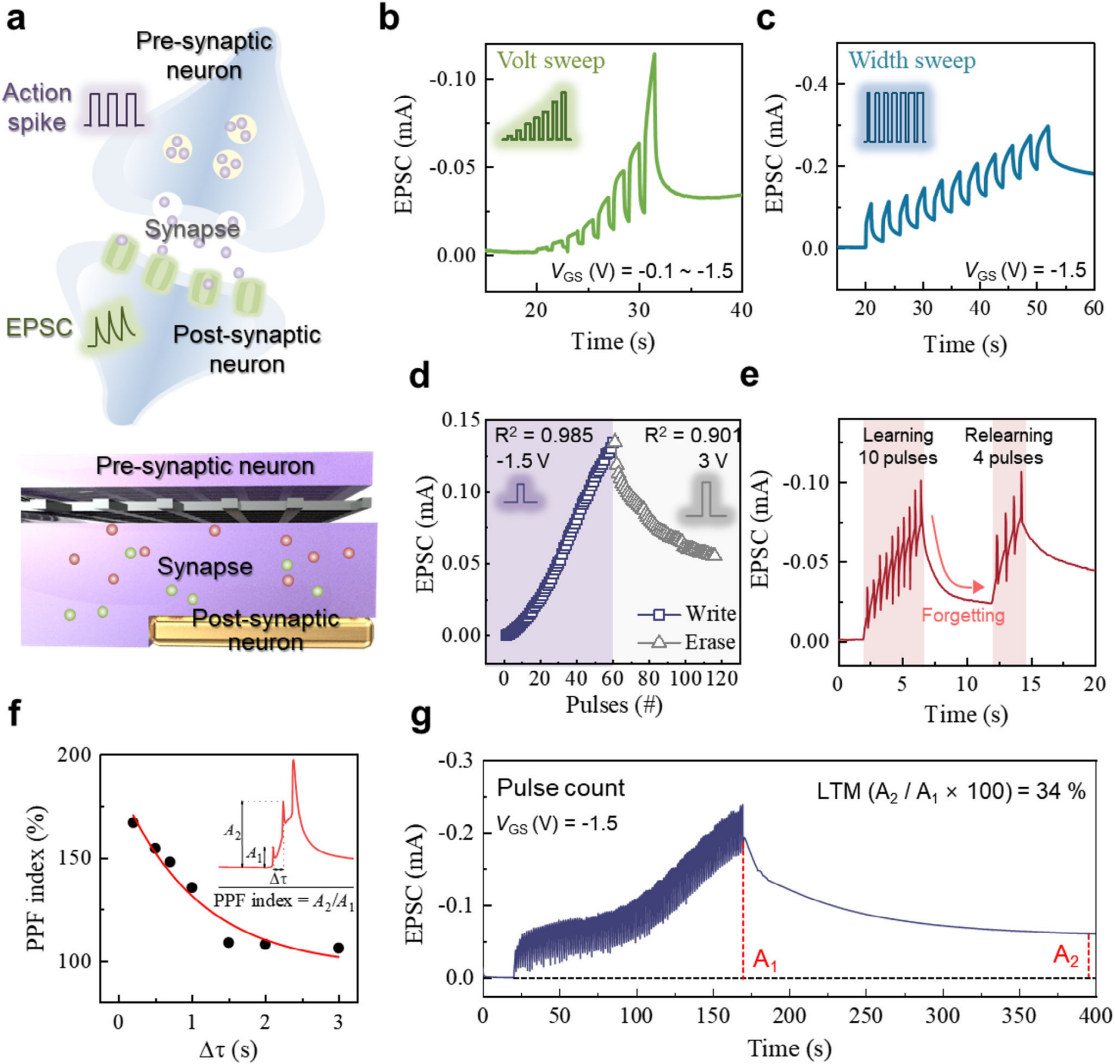
Synaptic behavior of π‐ion film/mesh OECT. a) Schematic illustration showing comparison of human synapse behavior and reinforced π‐ion film OECTs. Synaptic properties of π‐ion film/mesh OECT: b) Voltage sweep mode (*V*_GS_ from −0.1 to −1.5 V, step of −0.2 V and *V*_DS_ of −1 V), c) pulse width sweep mode (*V*_GS_ of −1.5 V, pulse width from 1 to 2 s and step of 0.1 s), d) write (*V*_GS_ of −1.5 V) and erase (*V*_GS_ of 3.0 V) curves, e) learning and re‐learning curve, f) PPF index and g) pulse count mode showing LTM property (*V*_GS_ of −1.5 V, *V*_DS_ of −1 V and 100 pulses).

Biological synapses play a crucial role in neuromorphic processes such as learning, memory, and information processing. Inspired by the functionality of biological synapses, neuromorphic devices were developed using π‐ion film/mesh OECTs. OECTs are particularly well‐suited for imitating synaptic behavior due to their ability to transport ions, which enables efficient doping mechanisms.^[^ ^44^, ^45^, ^46^ ^]^ This ion transport facilitates charge transfer and modulation of device conductivity, imitating the dynamic responses of biological synapses. The π‐ion film/mesh OECTs served as a compelling analog to biological synapses, as illustrated in **Figure** **3**a. In comparison, the *V*_GS_ pulses were applied to the devices to give the function like a pre‐synaptic neuron, while the resulting excitatory postsynaptic current (EPSC) represents the post‐synaptic neuron. The synaptic properties of the π‐ion film/mesh OECTs were evaluated through various modes of stimulation. In the voltage sweep mode, increasing the gate pulse intensity of *V*_GS_ led to a corresponding rise in EPSC and the baseline, clearly demonstrating synaptic behavior (Figure 3b). Similarly, when the pulse width was increased, the corresponding EPSC increased, and short‐term memory effects were observed upon pulse removal (Figure 3c). Figure 3d shows the write and erase properties in 50 pulses of π‐ion film/mesh OECTs showing memory capabilities, which exhibited high linearity (*R* ^2^) in the write mode of 0.985 and erase mode of 0.901. In Figure 3e, learning and re‐learning properties were shown in the condition where the devices were initially trained with 10 pulses, followed by a forgetting phase, and then re‐learned using just 4 pulses to reach the initial learning threshold, showing the memory properties of the neuromorphic devices. This confirms the synaptic characteristics of the device and its potential for neuromorphic applications. Furthermore, the paired‐pulse facilitation (PPF) index was calculated from the ratio of the second EPSC peak to the first EPSC peak showing 167% at a peak interval (∆τ) of 0.2 s (Figure 3f). For long‐term memory (LTM) analysis, after applying 100 pulses of the intensity of −1.5 V, EPSC was maintained at 34% after 250 s (Figure 3g). However, the devices showed relatively low LTM retention capability compared to conventional OECTs. Usually, the LTM retention capability of synaptic devices based on OECTs is functioned by materials such as poly(vinylidene fluoride‐co‐hexafluoropropylene), PVDF‐HFP, which are commonly used in the production of ion gels and have many F groups that can trap ions. Therefore, the LTM retention performance is bound to be lower than that of conventional OECTs produced with PVDF‐HFP‐based ion gels. In the future, we will produce a π‐ion film containing a material that acts as a trap, such as PVDF‐HFP, to implement a synaptic device with high‐performance memory retention capability in addition to the advantages of modularity and reusability in our current devices. When the same experiments were performed on pristine P3HT gel OECTs (Figure S14, Supporting Information), the low synaptic properties including low EPSC, baseline increase and low *R* ^2^ of 0.885 in erase mode were observed. These results indicated that the high capacitance of the π‐ion film (Figure 1f) enhanced ion transport and ion polarization, facilitating the synaptic behavior in the π‐ion film/mesh OECTs.

**Figure 4:**
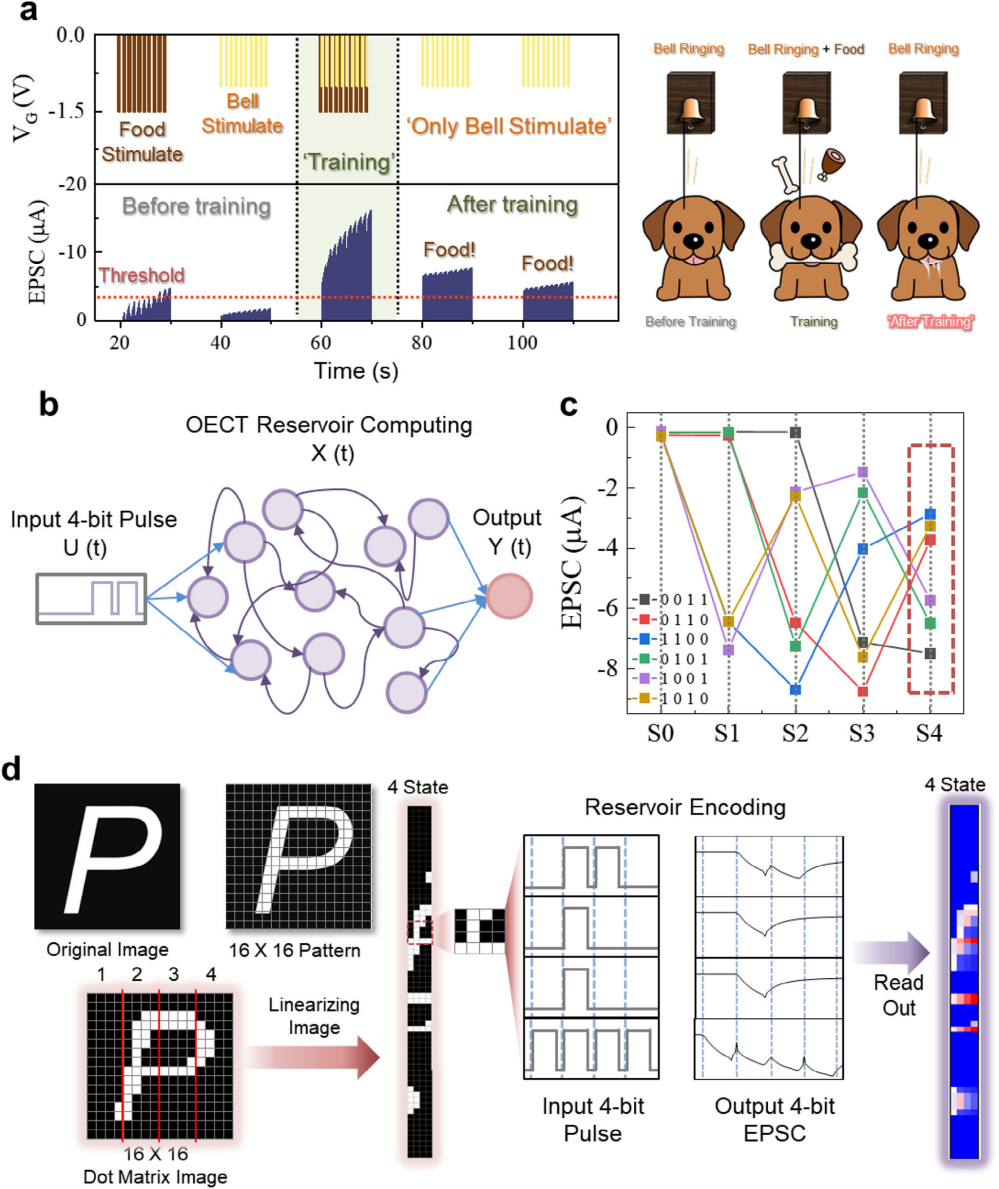
Applications of synaptic π‐ion film/mesh OECTs. a) Pavlov's dog phenomenon showing memory properties of synaptic devices. b) Reservoir computing system mechanism using π‐ion film/mesh OECT based on synaptic behavior. c) EPSC for each reservoir state of π‐ion film/mesh OECT according to representative 4‐bit electrical stimulus. d) Image reservoir encoding simulation. 4‐column combination of 16 × 16 patterns for input electrical stimulation (left) and analysis of the 4‐column pattern using EPSCs of a π‐ion film/mesh OECT‐based reservoir (right).

Based on the synaptic behaviors of π‐ion film/mesh OECTs, application to learning and re‐learning through Pavlov's dog simulation was studied (**Figure** **4**a).^[^ ^47^, ^48^ ^]^ In this process, a −1.5 V pulse indicated the food stimulus, while a −1 V pulse represented the bell stimulus. The EPSC value of 4.5 µA was defined as the threshold current of the dog's salivation response according to food stimulus synaptic weight (the red dotted line). Initially, only the food pulses were applied to strengthen the synaptic weight, giving a threshold standard. After a brief period of 10 s, applying the bell pulses alone did not exceed this threshold. When both food and bell pulses were applied simultaneously, the high EPSC surpassed the threshold. Then subsequent bell pulses alone after a period of 10 s could also exceed the threshold, confirming the learning effect of the devices.

Also, synaptic properties of π‐ion film/mesh OECTs can be effectively utilized within a reservoir computing framework.^[^ ^49^ ^]^ Synaptic behavior and reservoir computing were explored independently, each was operated in distinct modes of the π‐ion film/mesh OECTs. Synaptic behavior was demonstrated by applying repeated gate pulses to emulate LTM characteristics, such as conductance modulation and memory retention. In contrast, the reservoir computing experiment focused on short‐term memory operation by applying short‐duration, period gate pulses with defined timing intervals for the input signals. Reservoir computing utilizes the dynamic response of a system to process information. This system processes complex input signals through ion transport and charge distribution within the OSC channel, which acts as a “reservoir” providing nonlinear dynamic responses. These responses resulted in various reservoir states that can encode information in a way that captures complex temporal patterns. This mechanism was illustrated in Figure 4b, where the *V*_GS_ pulses are configured to generate a 4‐bit input, allowing for a total of 16 pulse input values (Figure S15a, Supporting Information). Each of these input pulses produced distinct EPSC responses, which were defined as reservoir states (Figure 4c; Figure S15b, Supporting Information) at *V*_GS_ of −1.5 V. Figure S15c (Supporting Information) shows that similar reservoir state trends were observed even when a low *V*_GS_ of −1.0 V was applied, with the smallest standard deviation of 0.0139 µA in the 0110 state and the highest standard deviation of 0.2023 µA in the 1111 state for five samples, showing the stability and reproducibility of the reservoir computing performance. Using the defined input pulses and reservoir states, the π‐ion film/mesh OECTs can be applied to image encoding. In Figure 4d, the letter “*P*” was transformed into a dot matrix image, slicing it into a 16 × 16 pattern. This pattern was linearized into 4 rows to create a 4‐bit input pulse signal. Following this process, the 4‐bit pulses were applied to encode the reservoir state values, allowing us to effectively read out the image data.

**Figure 5:**
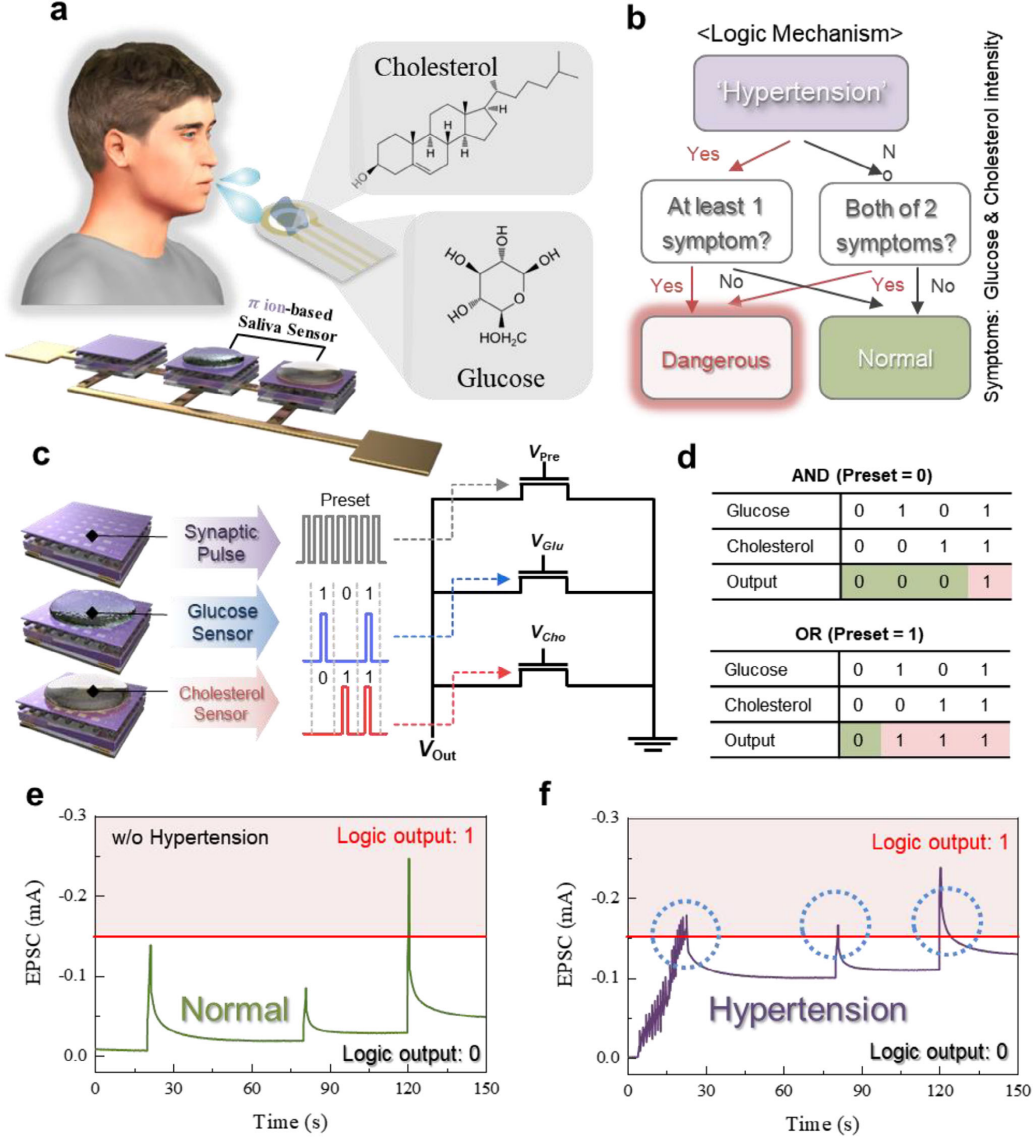
Suggested π‐Ion film OECT application for detachable logic sensor. a) Schematic illustration of the 3‐array logic bio sensor using π‐ion film/mesh OECTs. b) Logic mechanism for hypertension weights and symptom weights. c) 3‐array device structure showing pulse application for each device. The preset settings for synaptic pulses according to hypertension levels, and pulse applications based on glucose and cholesterol intensities. d) Results of AND and OR logic gates in response to pulse application, with normal (green) and dangerous (red) classification. Risk classification results based on pulse weights and logic for patients (e) without hypertension and (f) with hypertension.

Additionally, the potential application of π‐ion film/mesh OECTs as detachable logic‐based chemical sensors was conceptually proposed, particularly for bio‐sensing applications such as saliva analysis.^[^ ^50^ ^]^ The detachable feature of π‐ion film allows for sensor replacement after use, enabling sustainable and reusable sensor platforms. According to the logic gate simulations based on synaptic weights and the implementation of AND and OR logic gate mechanisms, bio‐logic sensors capable of assessing disease risk levels can be developed (**Figure** **5**a). The mechanism of the logic gate simulation as a bio‐sensor application was shown in Figure 5b, in which hypertension intensity is set to a preset intensity, allowing for the classification of risk groups or normal groups based on the presence of symptoms. If the patient has hypertension, only one symptom is required to be dangerous (OR logic); otherwise, both symptoms must be present to be dangerous (AND logic). The simulation of synaptic pulse inputs to a 3‐device array is shown in Figure 5c. In Figure 5d, the outcomes of the AND and OR logic gates were shown, indicating the generation of logic outputs when the threshold was exceeded. The EPSC results according to the synaptic weights for the normal group and risk group were shown in Figure 5e,f, which confirm the logic gate simulation as a bio‐sensor application in risk classification. The detachable π‐ion film platform can be used as such health monitoring kits due to the detachability to be easily replaced, giving reusability of the sensors. Although Figure 5e,f are simulation results illustrating the concept of logical bio‐sensor operation, we will conduct research focusing on an actual replaceable bio‐sensor in the future.

Also, the π‐ion film/mesh OECTs demonstrated significant potential for flexible applications, as evidenced by the bending test according to bending radius. As shown in Figure S16a (Supporting Information), electrical performance was measured under the various bending radius, revealing that even under strong bending conditions up to a 5 mm radius, the π‐ion film maintained high flexibility. The OM images of the bent π‐ion film were shown in Figure S16b (Supporting Information), confirming no structure breakdown showing the structural stability. The electrical measurements indicated the on‐current (*I*_on_) ratio during bending states (*I*_on,B_) compared to the flat state (*I*_on,F_), showing maintained electrical performance of OECTs based on π‐ion film after bending state (Figure S16c, Supporting Information).

## 3 Conclusion

In this study, detachable and modulable OECTs were successfully developed utilizing reinforced π‐ion films. The innovative design of these devices enhanced electrical performance, air stability and operation stability while providing the ability to easily replace and reuse the OSC layer, addressing key challenges in the field of organic electronics. Our findings demonstrated that the π‐ion film/mesh effectively supported the operational requirements of OECTs, leading to significant advancements in neuromorphic computing and bioelectronics.

The π‐ion film/mesh OECTs included a high *g*_m_ of 3.88 mS and µC^*^ of 206.80 F V^−1^ s^−1^ cm^−1^ due to the introduction of the π‐ion structure and conductive mesh support. Additionally, the devices exhibited impressive electrical characteristics, maintaining stable operation with an average µC^*^ of 131.32 F V^−1^ s^−1^ cm^−1^ even after cutting and detachment. The air stability of the devices also improved, allowing the devices to maintain over 95% consistent OECT behavior for 35 days in the air and up to 50 days during storage. The detachable and reusable characteristics of the π‐ion film with mesh directly can address the common issue of rapid degradation in conventional OECTs. This easy replacement of the π‐ion film can provide a wide range of applications for organic electronic products including the development of efficient and environmentally friendly device architectures through modular and reusable platforms. Furthermore, π‐ion film OECTs showed high‐performance neuromorphic characteristics and synaptic logic applications including Pavlov's dog phenomenon, reservoir computing, and logic biological sensing.

However, some limitations of this study must be overcome. Further optimization is needed to improve long‐term stability during the continuous detachment/attachment cycle, and exploring compatibility with different OSC materials is essential for broader applicability. Future research directions may include optimizing the device architecture for specific applications such as suggested logic biosensors and integrating OECTs into more complex neuromorphic systems will be crucial for advancing this technology. There is also potential for scaling up the fabrication process of the detachable π‐ion film, which could open commercialization opportunities for modular organic electronics. In addition, in this study, we fabricated the devices on a cm scale that can be controlled and picked up by humans for demonstrating detachability, replaceability, and modularity. Therefore, the large‐area integrated device will be realized in our future research by fabricating a large‐area π‐ion film and using a precise mechanical attachment and detachment method for the development from this study.

## Conflict of Interest

The authors declare no conflict of interest.

## Supporting information



Supporting Information

Supplemental Video 1

## Data Availability

The data that support the findings of this study are available from the corresponding author upon reasonable request.
