# Supplementary material for: Detachable and Reusable: Reinforced π‐Ion Film for Modular Synaptic Reservoir Computing
Source: Adv Mater. 2025 Jun 27;37(41):2506729. doi: 10.1002/adma.202506729 (PMC12531748; doi:10.1002/adma.202506729)
Supplement: Supplementary file 1 — Supporting Information [file ADMA-37-2506729-s001.docx]

**Supporting Information**

**Detachable and Reusable: Reinforced π-Ion Film for Modular Synaptic Reservoir Computing**

Gyu Won Woo*^1,^*†, Chang Min Lee*^1,^*†, Won Woo Lee*^2,^*†, Min Ju Jung*^1^*, Seung Min Lee*^1^*,
Hye Won Lee*^1^*, Hocheon Yoo*^3,^**, YongHee Kim*^1,^** and Eun Kwang Lee*^1,^**

*^1^Department of Chemical Engineering, Pukyong National University, Busan 48513, Republic of Korea*

*^2^Department of Semiconductor Engineering, Gachon University, Seongnam 13120, Republic of Korea*

*^3^Department of Electronic Engineering, Hanyang University, Seoul 04763, Republic of Korea**

Corresponding author

Email address: yonghkim@pknu.ac.kr (Y. Kim), hocheon@hanyang.ac.kr (H. Yoo), eklee@pknu.ac.kr (E. K. Lee)

*Keywords:* π-ion film, Organic electrochemical transistors, Detachable electronics, Synaptic devices, Neuromorphic reservoir computing

**Experimental Section**

*Materials*

P3HT was purchased from RIEKE metals. BMIM:TFSI, PVDF-HFP and polyvinyl alcohol (PVA) were purchased from Sigma-Aldrich. THF 97% was purchased from SAMCHUN chemicals. MeOH was purchased from Reagents DUKSAN. Stainless mesh was purchased from HIKARI. Au 3mmDia × 3mmTh (Au, 99.99%), Cr plated W rods (Cr), and Si 300 nm / SiO_2_ wafer were purchased from iTASCO. The chemical structures of materials were demonstrated in **Figure 1a**.

*π-Ion Film Fabrication*

**Figure S1** shows the detailed fabrication process of π-Ion Film. The glass substrates and meshes were cut into sizes of 2 cm × 2 cm. A solution of 15 mg mL^–1^ PVA of 250 µL was spin-coated onto the glass substrate at 1500 rpm for 30 s, followed by annealing at 110 ℃ for 1 min. The mesh was then placed on top of the glass substrate with the PVA layer. A circular mold with a diameter of 2.5 cm and a height of 1.5 cm was positioned inside a glass container, and the glass substrate with the mesh was carefully placed inside the circular mold. A polymer OSC solution was prepared by mixing P3HT and THF at a concentration of 10 mg mL^–1^, stirring the solution for 3 h. Then, 1 mL of the solution was carefully injected into the mold, and 3 mL of MeOH was sprayed around the mold, sealing the glass container for 24 h. Subsequently, 250 µL of BMIM:TFSI ion liquid was carefully injected into the circular mold, which was sealed again in the glass container for an additional 48 h to complete the fabrication of the π-ion film. After the π-ion film was formed on the glass substrate, the film was placed in DI water, resulting in the PVA layer dissolved in the DI water, separating the π-ion film with mesh support from the substrate.

*Device Fabrication*

To fabricate the reinforced π-ion film OECTs, Cr of 4 nm and Au of 40 nm source and drain electrodes with a channel width / length (*W* / *L*) ratio of 10 were thermally evaporated onto a glass substrate in vacuum condition under 10^–5^ Torr. The prepared π-ion film was carefully placed onto the electrode substrate, forming the channel.

To fabricate the P3HT spin-coated OECTs, 200 µL solution of P3HT was spin-coated onto a 2 × 2 cm glass substrate at 1500 rpm for 60 seconds. The resulting film was annealed at 130 °C for 30 minutes. Separately, an ion gel electrolyte was prepared by dissolving 0.5 g of PVDF-HFP in 10 mL of acetone, followed by mixing with 2 g of BMIM:TFSI. The mixture was stirred at 50 °C for 2 hours. After annealing the film, 1 µL of the prepared BMIM:TFSI electrolyte was drop-cast onto each channel, forming an ion gel state.

*Characterization Techniques*

The surface analysis and thickness measurement of the π-ion film were conducted using OM (OLYMPUS / BX53M). SEM (TESCAN / MIRA4) was employed to analyze the morphology of π-ion film fabricated on a SiO_2_ 300 nm / Si wafer. XPS (KRATOS Analytical Ltd. / AXIS SUPRA) was performed to analyze the uniformity and penetration effects of BMIM:TFSI, focusing on the C, O, N, and S element peaks. EIS (CORRTEST / CS100) was used to measure the capacitance of the π-ion film and BMIM:TFSI. Data was analyzed using appropriate software for peak identification and impedance modeling.

*Electrical Measurements*

All electrical performance measurements of the devices were conducted using the Keithley 2636A and Keithley 2636B Source Meter systems. The transfer curves were measured under *V*_DS_ of –1 V and a *V*_GS_ ranging from –3 to 1 V. For the output curves, the *V*_GS_ ranged from –0.4 to –2.0 V in steps of 0.4 V.

*Synaptic Behavior Analysis*

For the measurement of synaptic behavior, the Keithley 2636A and Keithley 2636B Source Meter systems were utilized. The measurement conditions are as follows: (1) voltage sweep mode with *V*_GS_ ranging from –0.1 to –1.5 V in steps of –0.2 V and a *V*_DS_ of –1 V. (2) pulse width sweep mode with *V*_GS_ of –1.5 V and varying the pulse width from 1 to 2 s in steps of 0.1 s. (3) pulse count mode to demonstrate LTM properties with *V*_GS_ of –1.5 V, *V*_DS_ of –1 V, and 100 pulses. (4) PPF index measurements with *V*_GS_ of –1.5 V, *V*_DS_ of –1 V, and 2 pulses with interval times ranging from 0.2 to 3 s. (5) write (*V*_GS_ of –1.5 V) and erase (*V*_GS_ of 3.0 V) curves with 50 pulses. (6) learning and re-learning curves, where learning was conducted with *V*_GS_ of –1.5 V, *V*_DS_ of –1 V, and 10 pulses, and re-learning with *V*_GS_ of –1.5 V, *V*_DS_ of –1 V, and 4 pulses.

*Reservoir Computing Simulation*

For the encoding of the letter “P” dot matrix image for the reservoir computing simulation, a 16 × 16 pattern was created, with areas filled over 50 % as white squares. The 4-bit input pulse condition was set at *V*_GS_ of –1.5 V, 1 s of pulse period and 0.8 s of pulse width as shown in **Figure S15**.


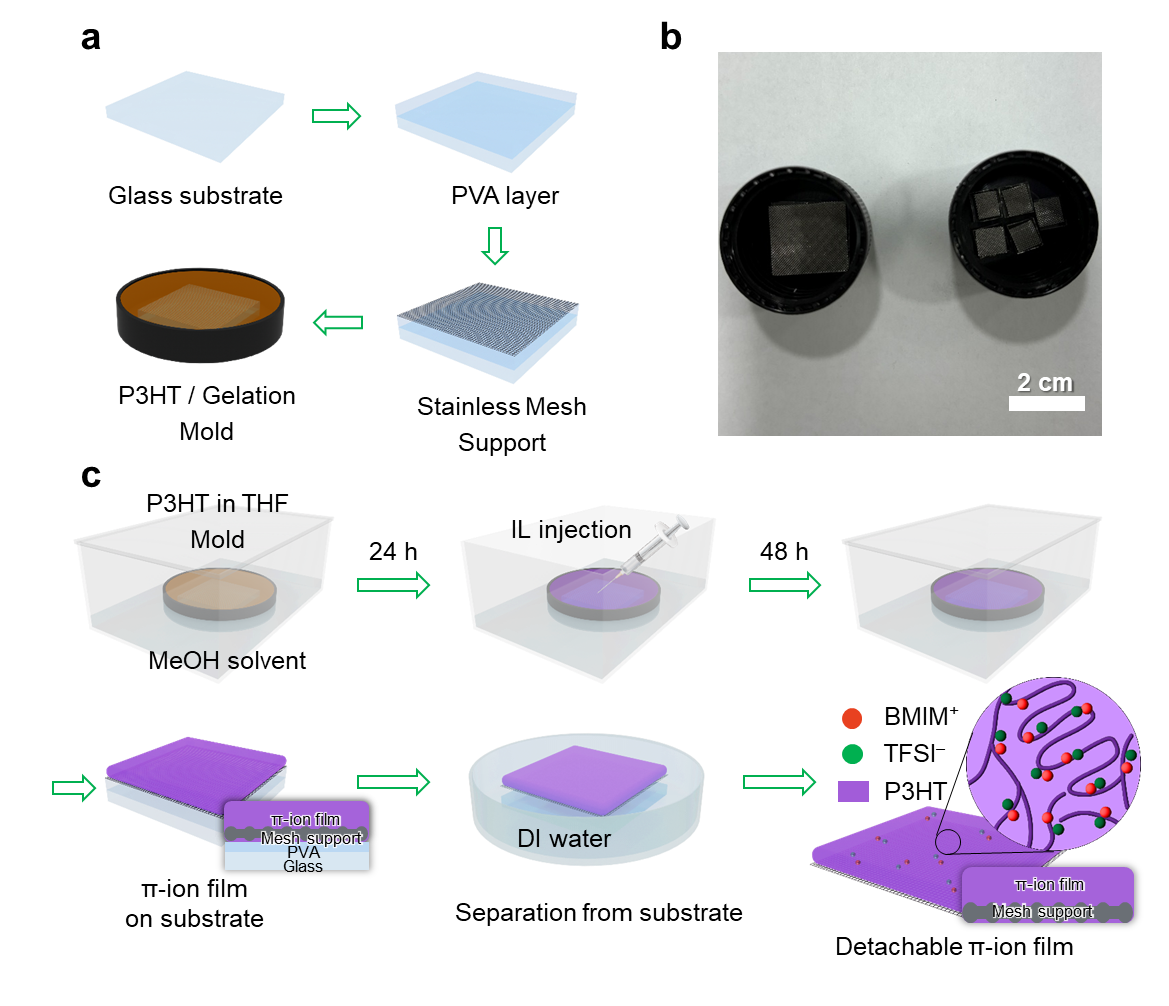


**Figure S1.** Materials and fabrication of π-Ion film. **(**a) Preparation steps of π-Ion film substrate and mold. (b) Photograph of preparation step without P3HT solution. (c) Gelation and separation steps of π-Ion film.


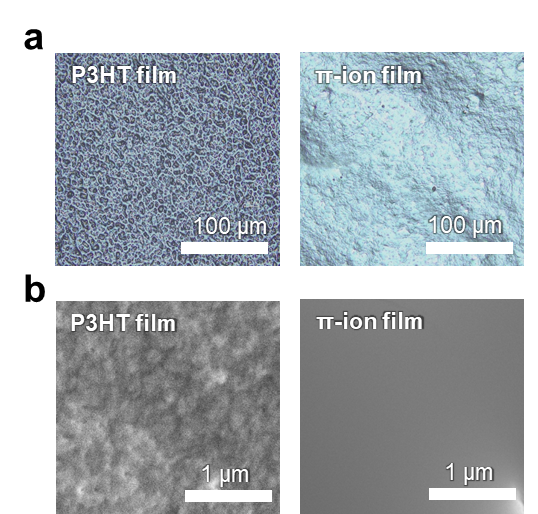


**Figure S2.** Surface analysis of π-Ion film**. (**a) OM images of pristine P3HT film (left) and π-Ion film (right). (b) SEM images of pristine P3HT film (left) and π-Ion film (right).


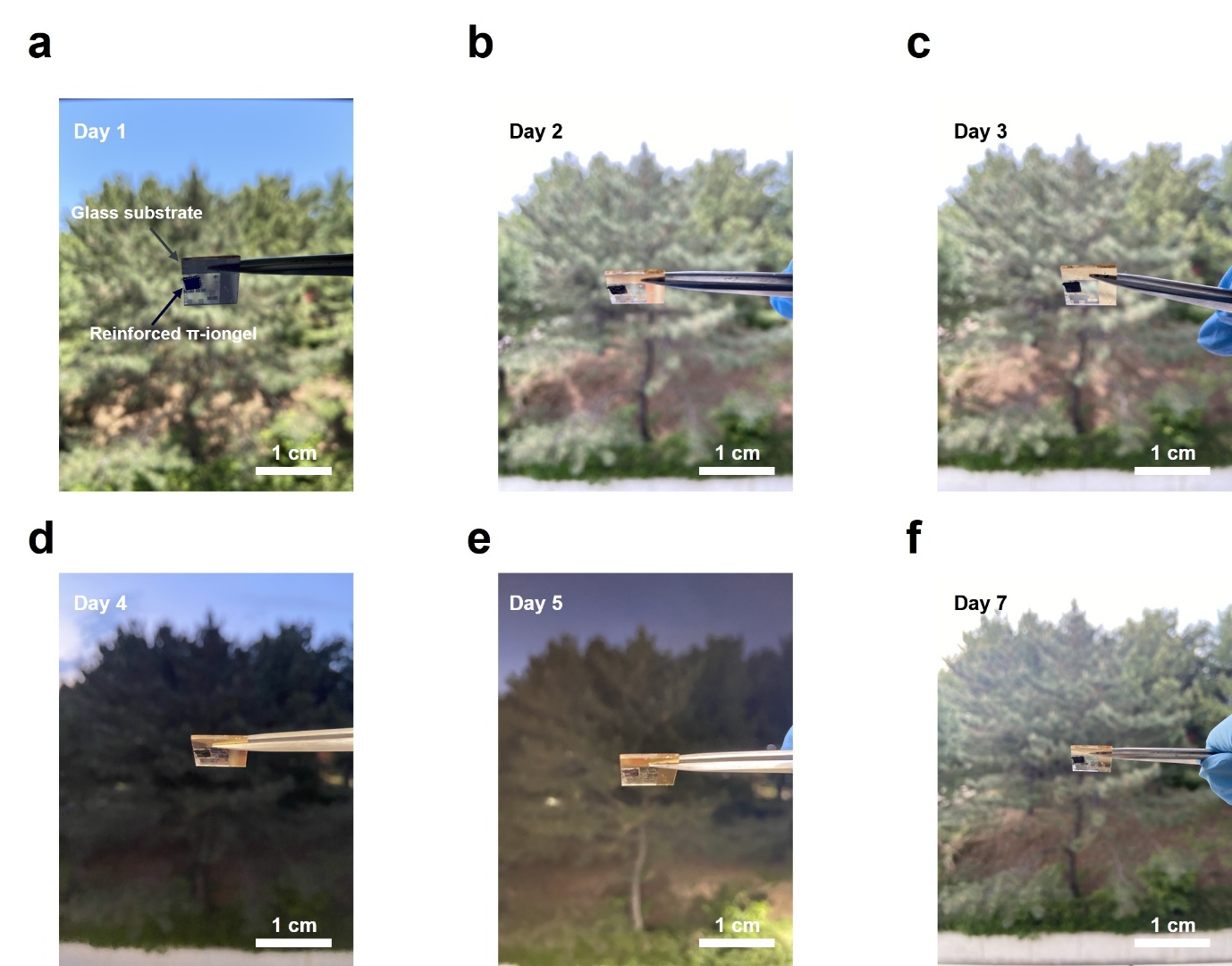


**Figure S3.** Adhesion characteristics of the π-Ion film on the substrate. Pictures of inverted π-ion film on substrate after (a) 1, (b) 2, (c) 3, (d) 4, (e) 5, (f) 7 days. The average temperature and humidity were 25 °C and 25%, respectively.


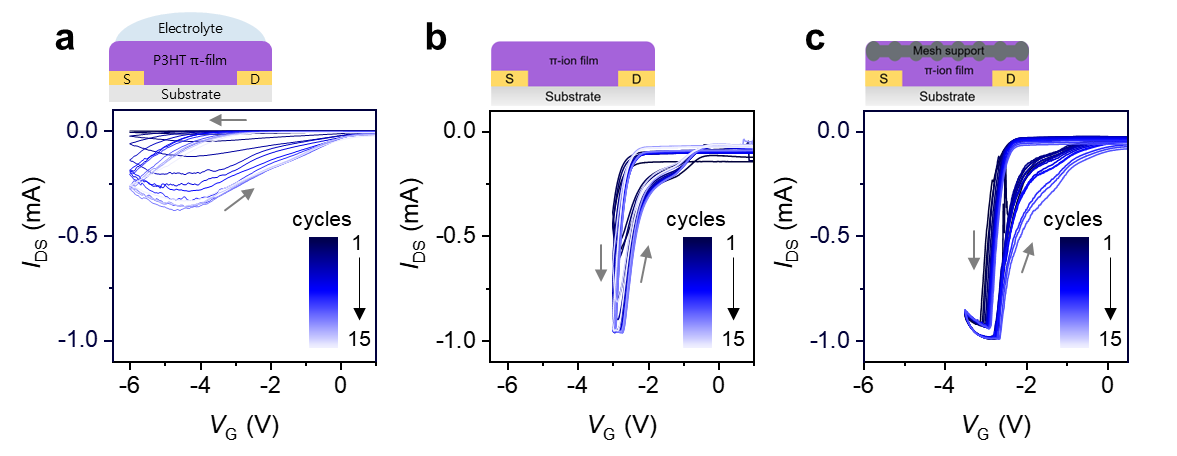


**Figure S4.** Electrical characteristics according to device structures**.** Transfer curves of (a) BMIM:TFSI electrolyte / pristine P3HT π-film, P3HT π-Ion film (b) without and (c) with mesh support (*V*_DS_ = –1 V).


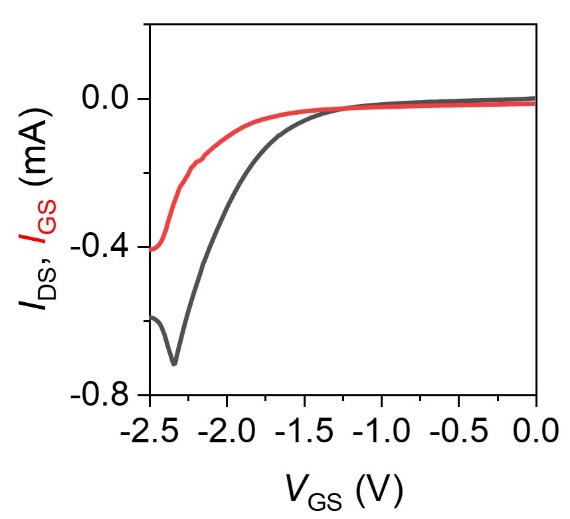


**Figure S5.** Gate leakage current of π-Ion film / Mesh OECT (*V*_DS_ = –1 V).


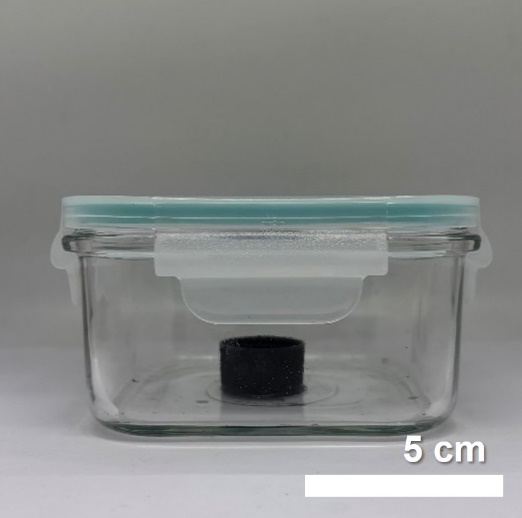


**Figure S6.** Photograph of π-Ion film storage at container for shelf-life stability test. Storage condition was temperature of 24.5℃ and humidity of 25%.


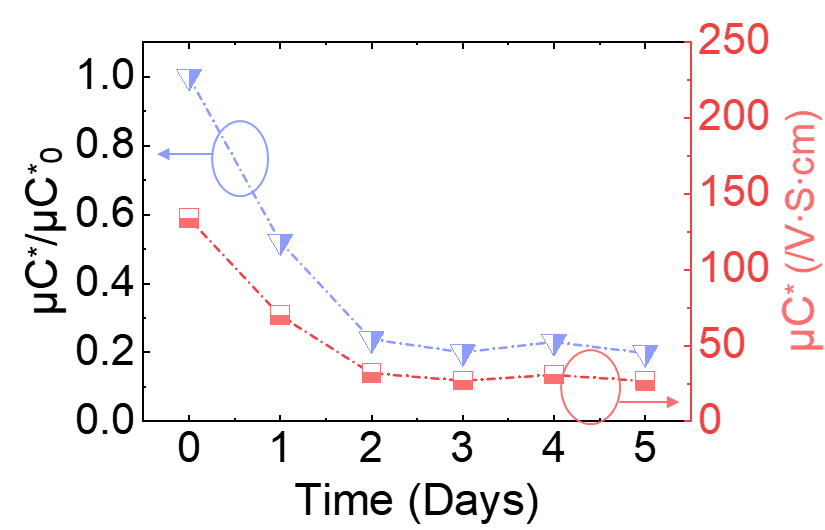


**Figure S7.** Air stability of spin-coated P3HT OECT with BMIM:TFSI electrolyte showing electrical performance degradation in ambient state (temperature of 25 ℃ and humidity of 25%).

Video

**Figure S8.** Electrical analysis of π-Ion film OECT: cutting and detachable experiments video.


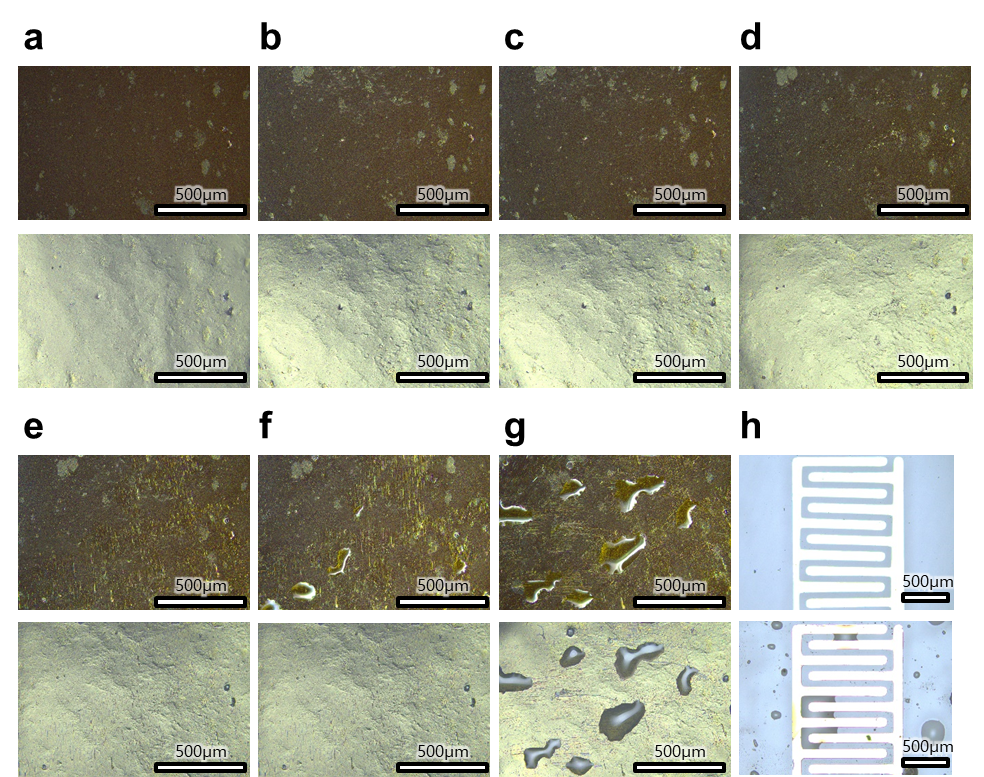


**Figure S9.** Detaching stability of π-Ion film. OM images of the π-ion film (top: dark-field; bottom: bright-field). Images from (a) to (g) correspond to the π-ion film after the 0th (pristine), 1st, 2nd, 3rd, 4th, 5th, and 6th detachment cycles, respectively. As detachment cycles increases, the surface roughness gradually increases from the 1st to the 3rd cycle. At the 4th cycle, visible scratches begin to form, and from the 5th to the 6th cycle, ionic liquid leakage from the gel matrix becomes evident. (h) OM images of the channel before attachment and after the 6th detachment. After the 6th cycle, leakage of ionic liquid and partial detachment of particles from the channel surface are clearly observed.


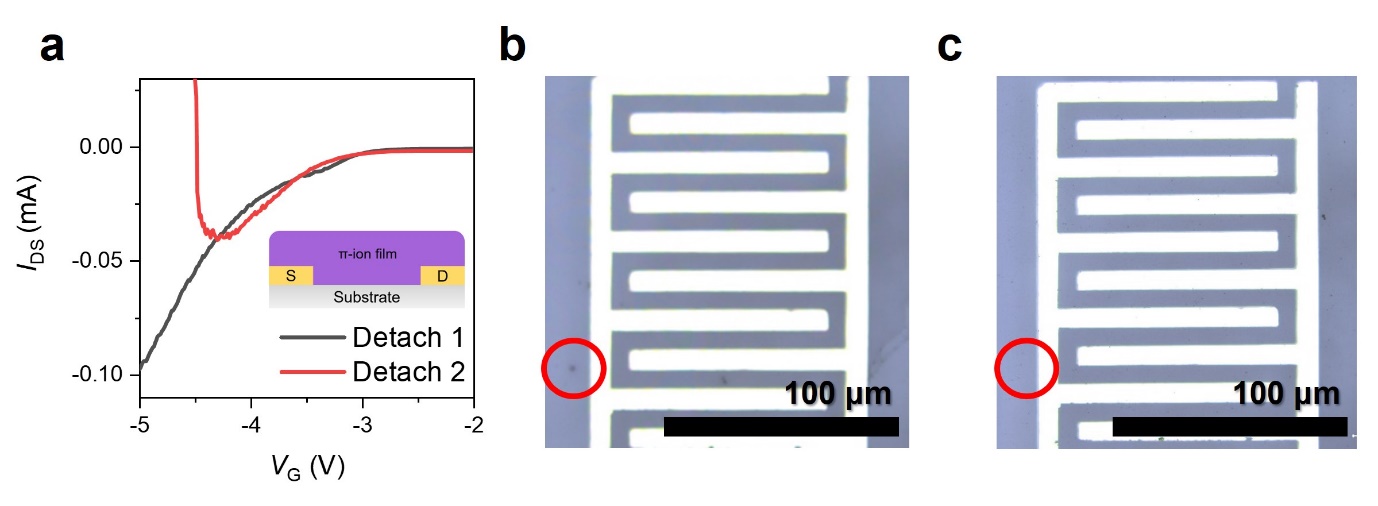


**Figure S10.** Detachability analysis of π-Ion film OECT. **(**a) Transfer curve of π-Ion film OECT without mesh support shows unstable performance (*V*_DS_ = –1 V). OM images for surface analysis before (b) and after (c) detaching the π-ion film. Particles initially present on the surface were removed during the detachment process.


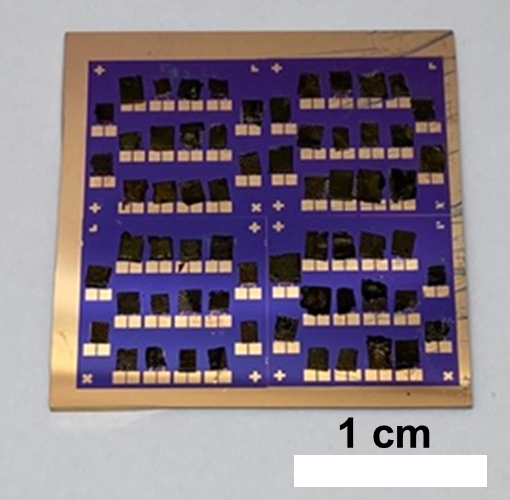


**Figure S11.** Photograph of large scale array of π-Ion film device.


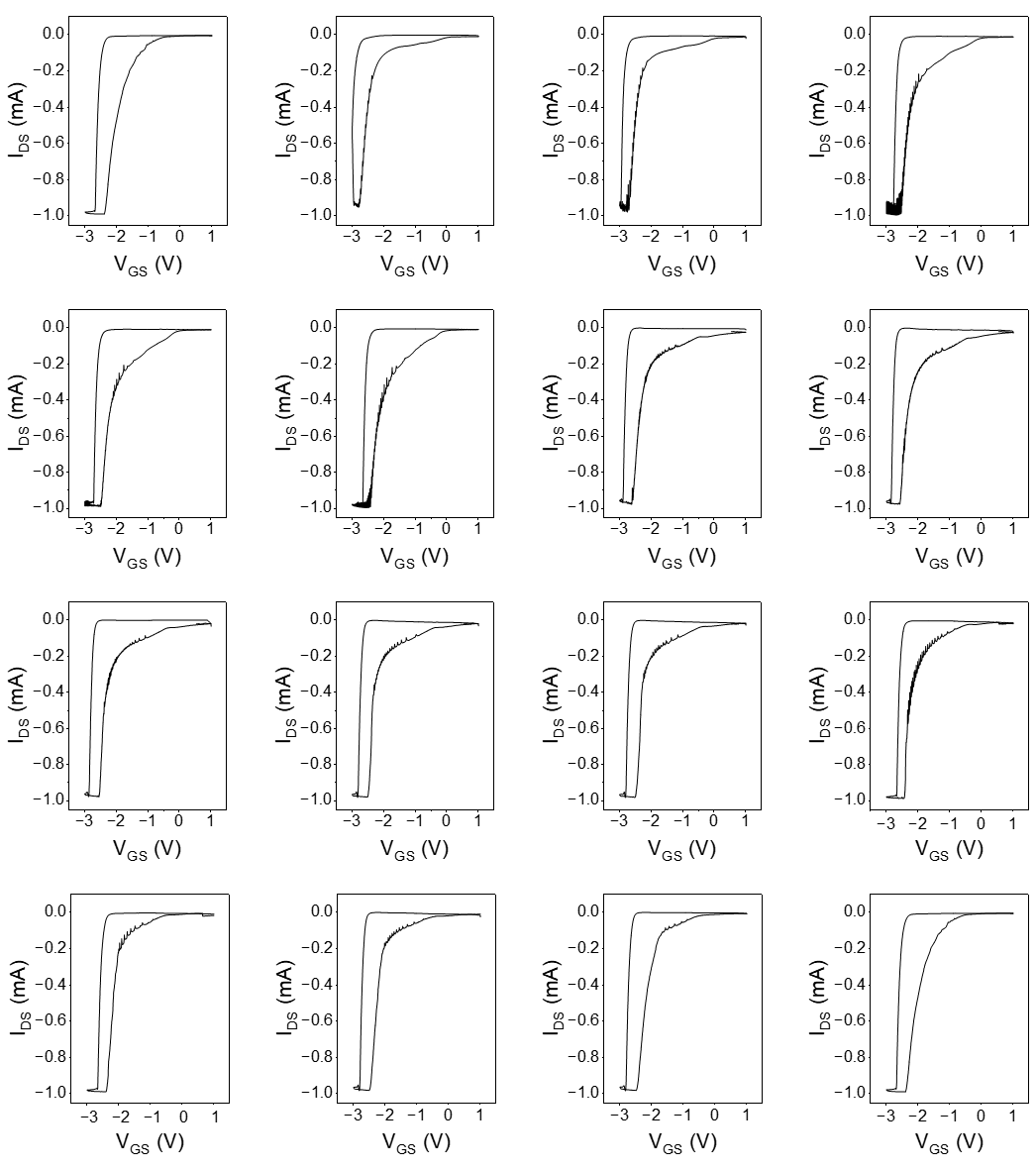


**Figure S12.** Transfer characteristics of large scale array of π-Ion film device.


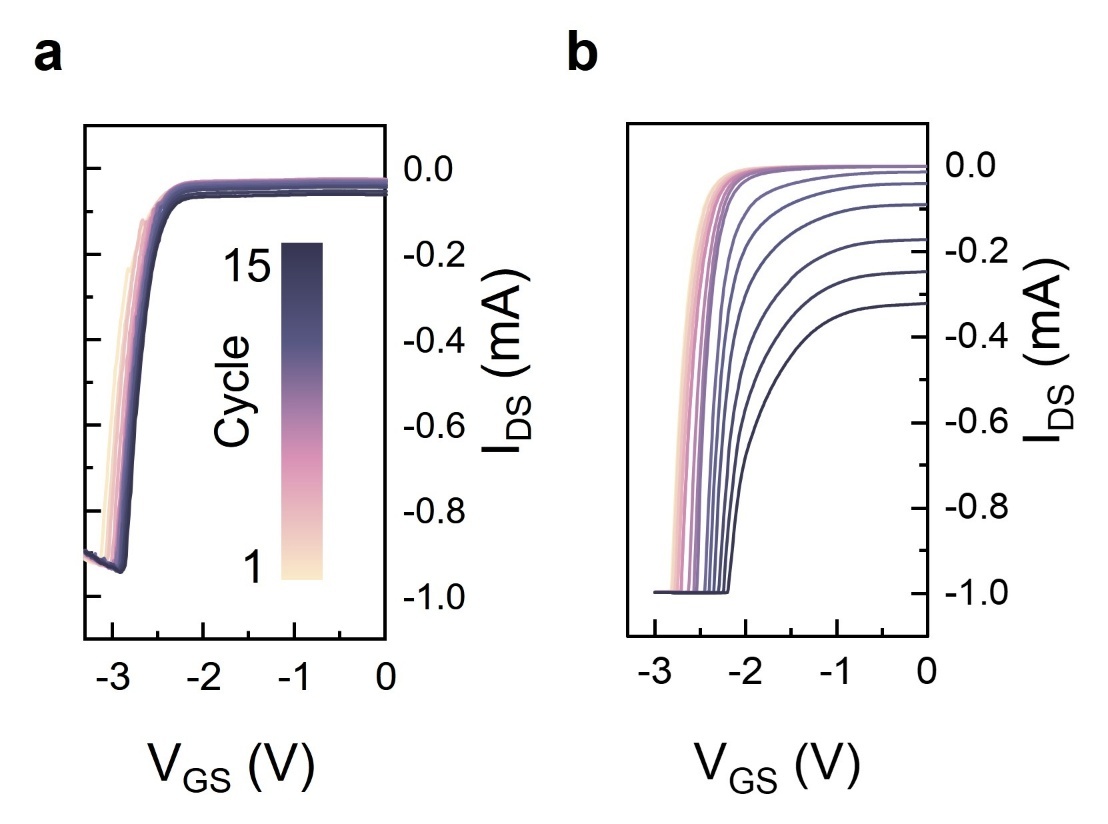


**Figure S13.** (a) 15 cycles transfer curves of π-ion film / mesh-based OECTs showing operation stability (*V*_DS_ = –1 V). (b) 15 cycles transfer curves of spin-coated P3HT OECT with BMIM:TFSI showing high hysteresis phenomenon (*V*_DS_ = –1 V).


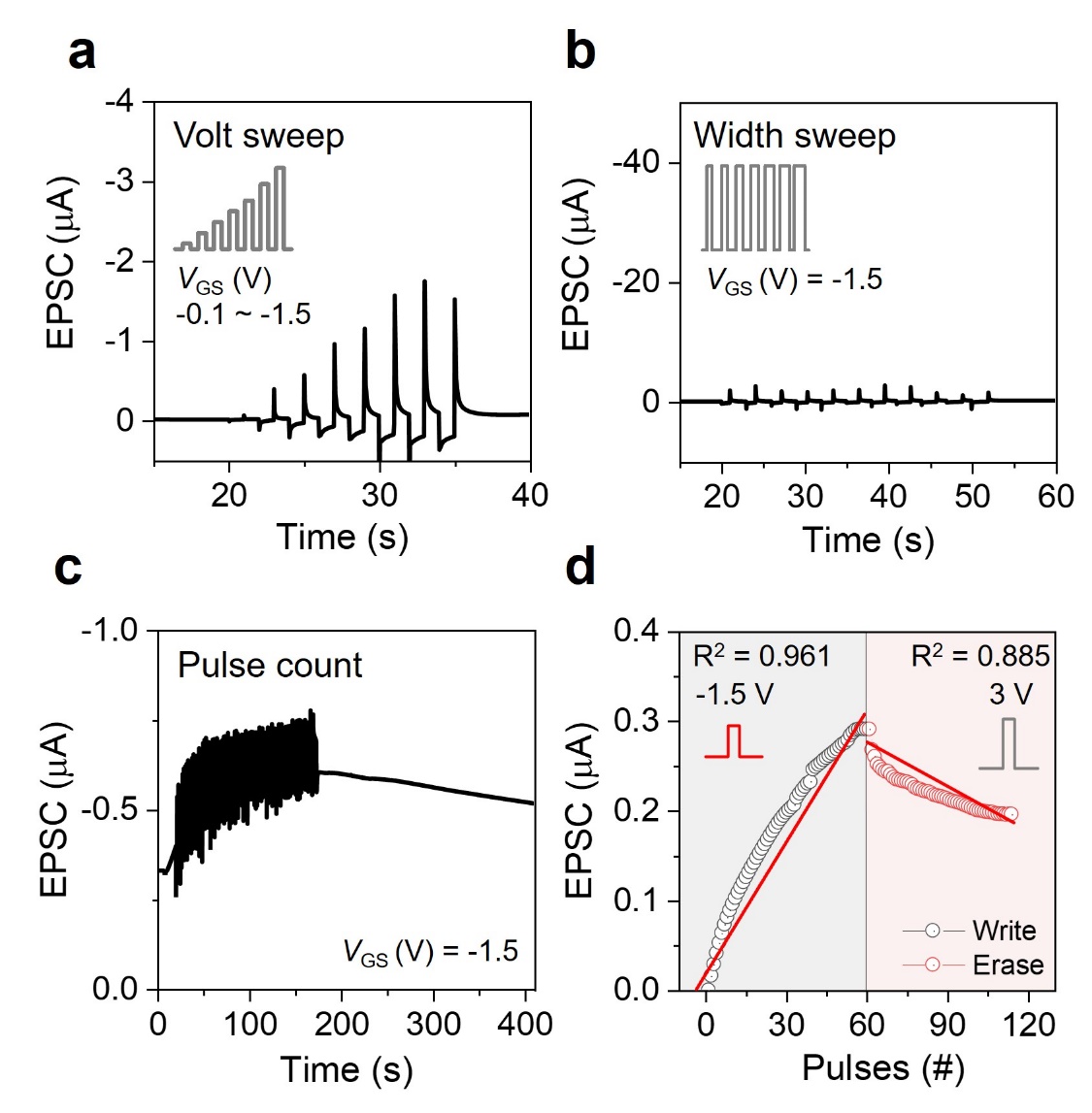


**Figure S14.** Synaptic behavior of pristine P3HT gel OECTs. Synaptic properties of P3HT gel OECT: (a) Voltage sweep mode (*V*_GS_ from –0.1 to –1.5 V, step of –0.2 V and *V*_DS_​ of –1 V), (b) pulse width sweep mode (*V*_GS_​ of –1.5 V, pulse width from 1 to 2 s and step of 0.1 s), (c) pulse count mode (*V*_GS_ of –1.5 V, *V*_DS_​ of –1 V and 100 pulses), and (d) write (*V*_GS_​ of –1.5 V) and erase (*V*_GS_​ of 3.0 V) curves.


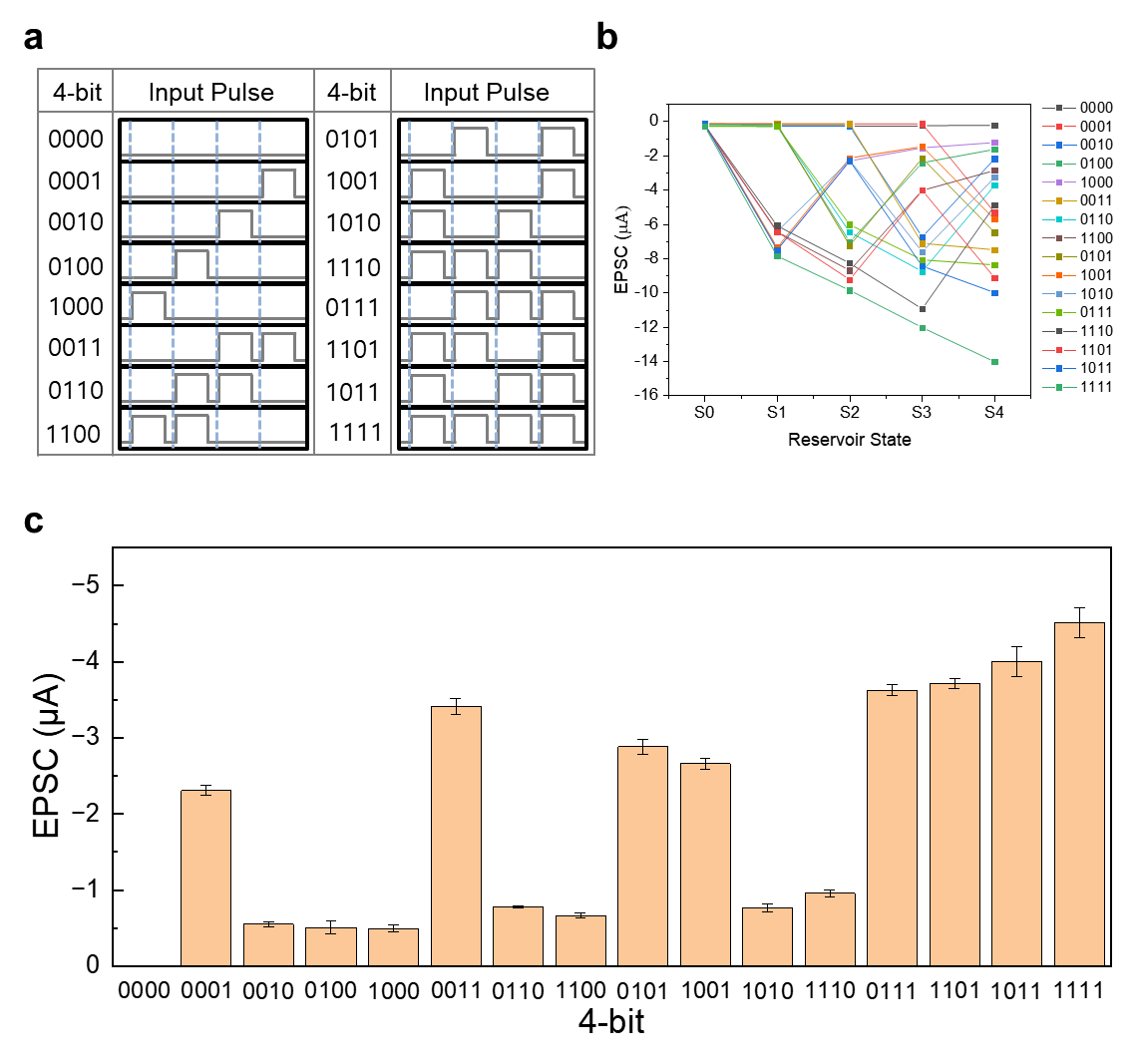


**Figure S15.** Synaptic OECTs-based reservoir computing. (a) Input gate pulse for each 4-bit. (b) Output reservoir state for each 4-bit (*V*_GS_ = –1.5 V). (c) Output reservoir states with error bars for each 4-bit input (*V*_GS_ = –1.0 V).


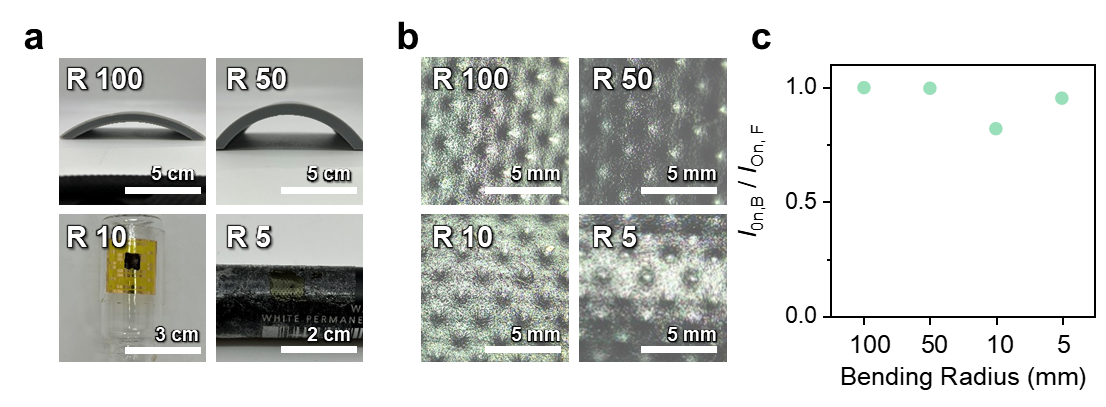


**Figure S16.** Flexible device application of the π-Ion film. (a) flexible state of π-Ion film according to bending radius. (b) OM images of π-Ion film at various bending radius. (c) *I*_on, B_ / *I*_on. F_ of π-Ion film according to bending radius.
